# Assessment of Medical Service Pricing in China's Healthcare System: Challenges, Constraints, and Policy Recommendations

**DOI:** 10.3389/fpubh.2021.787865

**Published:** 2021-11-26

**Authors:** Wenying Xiong, Yufan Deng, Yili Yang, Yumeng Zhang, Jay Pan

**Affiliations:** ^1^Sichuan Provincial Healthcare Security Administration, Chengdu, China; ^2^Healthcare Evaluation and Organization Analysis (HEOA) Group, West China School of Public Health and West China Fourth Hospital, Sichuan University, Chengdu, China; ^3^Institute for Healthy Cities and West China Research Center for Rural Health Development, Sichuan University, Chengdu, China

**Keywords:** medical service pricing, price setting, price regulation, health reform, China

## Abstract

Medical service pricing reform was considered as one of the focuses of China's remarkable health reform. This paper preliminarily assessed the roles of medical service pricing in the context of China's healthcare system. Specifically, we described the potential roles of medical service pricing in China and pointed out relevant challenges that emerged in practice as the result of reform-related activities. Multiple constraint factors that might have induced undesired outcomes were then recognized, including the excessive diversity and specialization of medical services, the price inelasticity of patients' demand, and the inadequate capability of both medical institutions and administrations. Finally, we provided policy recommendations to inform the ongoing medical service pricing reform in China from a long-term perspective.

## Introduction

Pricing policies are the key components of China's price management system, which are designed to ensure price stability as well as to balance the interests of all stakeholders engaged in the markets through price setting and regulation (1, 2). Since the foundation of the People's Republic of China, the promotion of a nationwide price management system has become a constant focus of China's national reform, with the overall reform process being divided into two major stages which were demarcated by the implementation of the reform and opening-up policy in 1978 (3). Exploring the appropriate degrees of authority delegation under a planned economy was the focus of the first stage, and the second stage that continued until this day aimed to establish a new price management system under a market economy, which was characterized by a market-oriented price formation mechanism with appropriate macro-control measures. During the second stage of the reform, the enforcement of the Price Law of China in 1998 served as a significant milestone in the development history of China's price management system (4), which managed to set up a list of fundamental rules and regulations for price acts under the socialist market economy.

As an essential part of national pricing policy, medical service pricing policy in China has gone through similar evolutions just as the whole price management system. In general, medical price management in China has evolved through three main stages: “strict regulation” (1949–1979), “decentralization” (1980–1999), and “authority delegation with proper regulation” (2000 onwards), with the form of medical service pricing evolving from government-determined price (administered price) in the first two stages, to government-guided price and market-regulated price in the third stage (5). The first stage was characterized by a consistent low-price policy, with public welfare being the focal point of medical service pricing. During that period, the Chinese government repeatedly implemented price reduction on medical services. For example, in 1956, the surgery fee and outpatient registration fee were decreased by about 60 and 30%, respectively (6). The second stage marked the initiation of authority delegation, during which distinct price variations began to emerge among medical service items in different types of medical institutions. Specifically, medical institutions were allowed to make reasonable price adjustment based on their medical infrastructure conditions and healthcare qualities. The third stage aimed to establish and improve a new medical price management system under the socialist market economy.

Under the guiding ideology of the Price Law of China, *National Health Service Price Items Standard*, which served as the regulation for medical service pricing, was enacted in 2001 (comprised of a total of 3,966 items) and was later amended in 2007 (4,170 items) and 2012 (9,360 items), respectively. By providing detailed elaborations on which medical service items can be charged as well as how their prices should be set, this document intended to regulate the price acts of all public and private not-for-profit medical institutions. For private for-profit medical institutions, they are loosely regulated by the document and are given certain degrees of autonomy. In general, the enactment of the *National Health Service Price Items Standard* promoted the standardization management of medical price acts, which laid foundations for the subsequent pricing reform in the nationwide healthcare system.

To some extent, medical service pricing policy was designed as an attempt to curb the soaring medical expenditures, which has remained a critical issue confronted by both developing and developed countries around the world (7, 8). From a global perspective, medical service pricing policies have been adopted by various countries as a policy instrument to improve medical expenditure control (9, 10), which would further protect patients from heavy financial burden posed by unaffordable medical cost, and ultimately achieve the worldwide penetration of universal health coverage (11–13). China's medical service pricing policy in the recent 20 years turned out to have the same pattern. Specifically, the Chinese government launched a major health reform in 2009 as an attempt to relieve the persistent issue of “*kan bing nan, kan bing gui*” (the medical service is hard to obtain while expensive), as well as to provide all citizens with equal access to basic healthcare with reasonable quality and sufficient financial protection (14–17). During the second phase of the reform (2012 onwards), medical service pricing was considered as one of the essential focuses for policy makers (18), and served as a breakthrough point in reducing medical expenditures. Despite that China has made substantial progress through the overarching reform efforts (19, 20), it should be noted that each single reform-targeted policy might only have limited effects toward the desired reform goals, or might even prove to be useless. As for medical service pricing, previous studies in the area of evaluating reform-induced outcomes provided controversial findings: while some studies confirmed that the desired reform goals had been successfully achieved (21–23), others indicated that the medical service pricing reform failed to yield satisfactory outcomes to facilitate medical expenditure control (24, 25). However, all the studies based on previous literature in this field have pointed out that a more in-depth pricing reform is urgently needed to tackle future challenges as well as to better serve the public.

Under such circumstances, the institutional reform of the State Council of China in 2018 was expected to shed light on the outlook of future medical service pricing in China. During the institutional reform, the Chinese government established the National Healthcare Security Administration (NHSA) as a functional department that incorporates both the medical price management system and the medical security system. It means that in addition to medical insurance and medical assistance, a list of other items was added into the range of responsibilities prescribed for the NHSA, including price setting and price regulation of medical services, medical consumables, and drugs, which all used to be under the management of China's National Development and Reform Commission. Such institutional transformation was expected to propel the progress of the pricing reform via turning the core management body into a more specialized sector.

This paper aimed to preliminarily assess the roles of medical service pricing in China within the entire healthcare system. Existing challenges along with their potential causes were recognized, and lessons learned from the pricing reform were further discussed. Our study would contribute to the current literature from two aspects: First, we briefly depicted an overview of medical service pricing in China to inform future research in this field. Second, we provided policy recommendations that could be used to inform future reforms in China down the road, as well as for other countries confronted with similar situations.

The whole paper was composed of five sections. The first section briefly introduced the development of the medical price management system in China. The second section identified the potential roles of medical service pricing in China's healthcare system. The third section assessed medical service pricing throughout China's health reform in terms of the challenges and undesired outcomes induced by price-related policies. The fourth section intended to point out the potential constraints previously posed on medical service pricing. In the last section, we proposed policy recommendations for future medical service pricing reform in China based on our findings.

## Potential Roles of Medical Service Pricing in China's Healthcare System

Price is the financial amount that a purchaser pays to a provider to deliver a service. It is the value of goods (service) expressed in money or the momentary expression of value. For most products and services in the general market, price is formed through market competition and acts as a signal of supply-demand relationship. Unlike conventional markets, the healthcare market is characterized by several features, such as a high degree of information asymmetry and a monopolistic competitive market (26), which might consequently compromise self-regulation and resource allocation. In consideration of the essential role of healthcare system in both national economic development as well as in the aspect of maintaining residents' quality of life, healthcare services in China have long been under the regulation of the Chinese government. In the field of medical service pricing, such management strategy is manifested by a government involvement in price setting and regulation process.

Concretely speaking, an unbundling approach is currently adopted by the Chinese government for medical price setting: (a) unilateral administrative price setting (government-guided price) for basic medical services in public medical institutions; (b) individual or collective negotiations (market-regulated pricing) for special medical services in public medical institutions, which are either confronted with considerably competitive markets or are delivered in a highly personalized manner; (c) individual or collective negotiations (market-regulated pricing) for medical services in private medical institutions (27). As the guiding principles for medical service pricing, the *National Health Service Price Items Standard* explicitly stipulates which services can be charged, and how prices should be set for all public and private not-for-profit medical institutions. Theoretically, private for-profit medical institutions are unconstrained by these documents, which means that these institutions are allowed to provide medical services outside the range of the item list with self-determined prices. Nevertheless, in order to have the eligibility as medical insurance designated hospitals as well as to enhance their competitiveness within hospital markets, these institutions tend to comply with the standard to a large extent, which therefore ensured the penetration of such standard in China's healthcare system. In the context of China's healthcare system, the potential roles of medical service pricing are summarized as the following policy goals.

### Interest Coordination as an Embodiment of Public Welfare

Healthcare is an affair concerning public welfare, with its basic value being guaranteeing the public interest. Under the widely known political slogan “Serve the people,” health reform in China pledges to provide all citizens with equitable and accessible medical services, which reflects the people-centered orientation of the Chinese government. As a commonly adopted policy instrument, medical service pricing possesses distinct ideological significance (28). The interest coordination function of medical service pricing helps to stabilize the overall price level of medical services and to reduce the financial burden of patients, which therefore serves as a concrete embodiment of international consensus to achieve universal health coverage.

### Signals to Guide Resource Allocation

Prices serve as effective signals to inform the optimization of resource allocation for all kinds of social resources. As for medical services, the pricing largely determines the amount of social resource distributed among various health sectors, while also affects the allocation of resources among medical institutions at different levels as well as among medical services of different kinds. The former affects allocative efficiency, that is the strategic position of health sector in the process of socio-economic development, while the latter influences the productive efficiency of the healthcare system.

### Compensations for the Cost of Medical Service Provision

Medical service pricing also has a vital function in financial compensation. China has witnessed a soaring health expenditure during the past decades, with the total health expenditure (THE) exceeding 6.4% of the national GDP in 2019. Although the government has made massive investments in health sectors since 2007, it remains a tough task to compensate for the cost of medical service provision merely through governmental financial input. Despite the initial rising from 2007 to 2010, the share of THE by government health expenditure (GHE) presented to be stable around 30% in the subsequent years, which then began to show a downward trend since 2016. In 2019, GHE, social health expenditure, and private health expenditure accounted for 27.36, 44.27, and 28.36% of THE, respectively (29). Since the government took “*demand-side up* (*Bu Xu Fang*)” as the policy orientation (more government investments to subsidize the basic health insurance programs in the demand side, rather than the medical institutions in the supply side) in the new round of China's national health reform, medical service pricing has become the most important approach to make financial compensations for medical institutions. Rational medical service pricing ensures that medical institutions are adequately reimbursed and could make reasonable profits.

### Incentives for Health System Objectives

Another critical function of medical service pricing is to serve as financial incentives to achieve certain healthcare system goals, which plays an important role in both demand and supply sides. For examples, on the demand side, patients could hardly make optimal choices due to the highly specialized nature of medical services (30). Rational medical service pricing will guide patients' value discovery, and promote their rational health-seeking behaviors. Patients will be motivated to make choices with higher cost-effectiveness (or cost-utility) based on actual conditions, such as seeking medical services from medical institutions at appropriate levels as well as choosing medical services of appropriate types (31). On the supply side, medical service pricing will affect the service provision behaviors of medical institutions. Specifically, rational medical service pricing has great potential to inspire medical institutions to provide more valuable services (probably through technological innovation), as well as to improve the accessibility of specific services delivered to specific population groups (such as pediatrics and psychiatry).

## Challenges of Medical Service Pricing During China's Ongoing Health Reform

In-depth medical service pricing reform has been a key component of health reform in China. Although significant breakthroughs have been made in abolishing the profit-making mechanism of public hospitals, unintended outcomes due to policy flaws were also identified, which indicated that the potential policy goals of medical service pricing were not fully achieved (32, 33). We recognized the existence of these following challenges embedded in the implementation of the reform.

### Large Variations in Cost-to-Price Ratio Among Medical Service Items

The prices of medical service items remained roughly steady in China in the past decades. However, the cost of medical service provision, which is largely determined by the whole society, constantly changes along with socio-economic development. Therefore, a relatively stagnant pricing policy would possibly result in great differences in the amount of financial compensation (cost-to-price ratio) made for different types of medical service items (34). Profiting or even making ends meet would be challenging for medical services that were not adequately compensated (e.g., blood routine tests) in some medical institutions.

Disparities in financial compensation of different medical service items directly affected the provider's behaviors, which led to both shortage and surplus of certain medical service items. Private medical institutions in China served as a good example to describe such phenomenon: most of the private medical institutions in China were specialized hospitals that provided low-risk and high-profit services such as cosmetology and cardiology (35). Such phenomenon was found to be equally prominent in public medical institutions. Specifically, public medical institutions tended to expand departments with high-profit services (e.g., oncology), and to restrict those providing relatively low-profit services (e.g., pediatrics). For example, from 1996 to 2018, the number of national hospital oncology beds increased by 428%, from 43.3 thousand to 228.5 thousand, while the growth rate of hospital pediatric beds was reported as merely 150%, from 138.7 thousand to 346.9 thousand (29).

### Inadequate Compensation for Medical Staffs via Medical Service Pricing

Healthcare system is a labor-intensive industry, in which most services are delivered by medical staffs. However, the price of medical services involving technical work, such as diagnosis, surgery, and nursing, remains low for a long time (36). The situation has been improved since the launch of medical service pricing reform, with the price of medical services including nursing and surgery being adjusted upward. Meanwhile, the wage level of medical staffs was further raised after the implementation of the “*two permissions*” policy in 2017, a salary system reform in public hospitals instructed by Chinse President Xi Jinping. From 2010 to 2018, the proportion of staff costs among total costs has increased from 24 to 34% for all public hospitals (29).

Despite such a substantial increase in the share of labor-related compensation as a part of the total hospital expenditure through the reform effort, a wide gap still existed between China and developed countries in this regard, indicating the room for further improving the currently implemented medical service pricing policies. For example, the share of labor-related compensation as part of the total hospital expenditure among not-for-profit hospitals in the U.S. was reported as 50.6 and 54.9% in 2008 and 2018, respectively (37), which was well above that of all public hospitals in China. In addition to relatively inadequate compensation made for healthcare-related human resource, the value of technical work with different difficulty levels was also poorly recognized, thus indicating the necessity to further optimize medical service price setting procedures to better reflect difficulty levels in the future.

### Tiered Pricing Approach Posed Limited Influences on Patients' Health-Seeking Behaviors

The tiered medical service pricing approach (government-guided price based on the level of medical institutions) currently adopted by the Chinese government aimed to support the development of a hierarchical medical system. It tried to optimize the patient flow among different levels of medical institutions via improving patients' rational health-seeking behaviors. However, due to a list of flaws potentially embedded in such approach, it failed to have desired leverage effects on guiding patients' health-seeking behaviors. As such, establishing a rational health seeking pattern from a holistic perspective remains a long-term task in the context of China's healthcare system (38).

### Medical Service Pricing Related Health Reform Affected Provider's Financial Performance

The public hospitals in China used to be allowed to add a 15% profit margin for drugs to make up their cost in service provision. To address the problem of over-prescription, the zero make-up drug policy has been implemented since 2017. After it, the profit margin of drugs was removed. To compensate the loss of the hospitals, the government has consequently raised the prices of a part of medical service items.

The increases of prices have been set approximately according to the total loss of hospitals at the city level. However, due to variations in medical practices, the prescription amounts differed among medical institutions, resulting in different losses or benefits due to the policy reform. Different medical institutions responded with different coping strategies, and ultimately demonstrated different financial performances. As the result, some medical institutions managed to make ends meet or even achieve an income growth, while others failed to strike a balance between income and expenditures, thus facing varying degrees of surplus changes (39).

Similar results were found when it came to medical departments within a single medical institution: departments that once relied on drugs as the main source of surplus would suffer from the policy, while those that previously had made surplus via the services with the raising prices would benefit from these policies. In response to the policy changes, some medical institutions tackled the financial challenge at the institutional level, through distributing the overall surplus based on the actual workload of each single department. This avoided undesired financial performance gap among different departments. Others transferred the financial challenge directly to each department via determining each department's surplus based on their own financial performance, which as the consequence induced a larger income disparity after the pricing reform.

The recent policy reform, such as zero make-up consumables policy also similarly influenced the behaviors and financial performance of the supply side via medical service pricing.

## Constraints on Medical Service Pricing in China's Healthcare System

The function of medical service pricing has been improved gradually through the development of the medical price management system. As the health reform in China went into a deep-water zone, medical service pricing reform should also be adjusted accordingly. At the new stage of China's national health reform, we identified a list of potential factors which might pose negative impacts on the performance of medical service pricing.

### Highly Specialized and Diversified Nature of Medical Services

The current prices of medical service items have been set mainly based on cost in China. The highly specialized nature of medical services exacerbates the information asymmetry persistently embedded between service providers and administrative organizations, which as the consequence compromised the rationality and feasibility of cost accounting. Even if the administrative organization estimates the labor and material input at each step of service delivery microscopically, a detailed measurement approach won't necessarily produce accurate outcomes. There are several causes that might lead to inaccurate cost accounting outcomes. First, the steps of service delivery used in cost accounting methods might not be consistent with those in the real world. Second, the lack of consensus on diagnosis and treatment standards for some medical service items would add difficulties to produce cost accounting outcomes that are widely applicable. Costs might vary across medical institutions due to variations in medical practice. Third, apart from the variable cost of medical services, the administration cost should also be considered during cost accounting process, which usually varies among institutions. Fourth, medical service pricing would affect the service-providing behaviors of medical staffs, which could in turn influence the cost. Indeed, actual medical service pricing partly depends on the game between administrative organizations and service providers, and in most cases, cost accounting only serves as an endorsement of the results.

A high level of diversity should be noted as another challenge in medical service pricing. The current version of the *National Health Service Price Items Standard* contains 11 main categories with 9,360 different items. Such a wide range of medical service items has posed great pressure on administrative organizations, and this problem is very likely to be further intensified with the emergence of new medical technologies.

### Price Inelasticity of Patient's Demand for Medical Services

For most products (commodities and services) in the general market, price influences the behaviors of both supply and demand sides to achieve productive and allocative efficiencies of the market. Price elasticity is a measure of how sensitive the quantity demanded or quantity supplied is due to a change in price. It should be noted that at the individual or household level, patient's demand for most medical services is price inelastic, which means that its price elasticity is <1 (40). The patient's demand for medical services is poorly responsive to price, and the change in quantity demanded is rather small whether the price goes up or down. Due to all these underlying mechanisms, medical service pricing would pose limited effects on the demand side. Such effects would be further weakened by patient's poor health literacy or inadequate health-related information disclosure to the public.

### Insufficient Management Capacity of Medical Institutions

As stated in the previous section, different medical institutions demonstrated large variations in their financial performances in response to the medical service pricing related health reform, which reflected varying levels of management capacity. On the one hand, “reducing patients' financial burden” has been deeply rooted within each round of health reform, in which the people-centered orientation has been persistently highlighted as an essential goal. As such, medical institutions, especially those in public sectors, should be aware of their responsibilities to achieve those policy goals proposed for the health reform, and respond to the policies adjustments proactively by optimizing their internal operation systems. On the other hand, management capacities in multiple aspects, including human resource management, performance assessment, and cost control, should be emphasized as key determinants of whether a medical institution could effectively respond to a series of changes induced by the medical service pricing related health reform. As discussed, many medical institutions have transferred financial challenges directly to individual departments, which reflects the universal existence of a flawed management system within medical institutions. Therefore, medical institutions are in the urgent need to enhance their internal management, which has the capacity to support the normal functioning of the medical service pricing approach.

### Inadequate Governance Capability of Medical Service Pricing Administration

Although medical service pricing only has limited impacts on the demand side due to the price inelasticity of patients' demand, this is not the case for the supply side. The medical service provision is sensitive to the medical service price, which is reflected by the existence of both shortage and surplus in the provision of different medical service items. To effectively utilize medical service pricing as an incentive instrument, the governance capability of medical service pricing administration should be strengthened. Currently, the inadequate governance capability of NHSA has been reflected in multiple aspects.

First, the staffing of NHSA is hardly sufficient to accomplish a series of challenging price management tasks involved in medical pricing procedures. Taking Taiwan and Sichuan provinces of China as an example, the Health Insurance Administration (HIA) in Taiwan province is staffed with over 6,000 employees, serving a population of 23.6 million. In sharp contrast with Taiwan province, the total number of staff equipped for NHSA at all administrative levels in Sichuan province, a province with a significantly larger population of 83.75 million residents, fails to reach 3,000, which is much lower than half of the staff equipped for HIA in Taiwan province. Although the scope of responsibilities of NHSA staff and HIA staff might differ from one another, a nearly 7-fold disparity in the number of staff is daunting enough to alert the critical understaffing issue currently embedded in NHSA.

Second, the whole process of medical service pricing needs to be further improved under the supervision of NHSA. This could include the construction of a well-functioning interest expression mechanism for all stakeholders involved, the establishment of an effective accountability mechanism, and the formation of a list of evaluation criteria for assessing outcomes induced by price change policies (5).

Third, with the development of data science, the governance capability of NHSA is expected to be greatly enhanced by the adoption of big data analyses, which, however, requires a well-established nationwide information management platform for medical service prices. The current “information island” embedded in the healthcare system makes it impossible for NHSA to conduct a series of price adjustment tasks such as comprehensive monitoring, effective evaluation, and dynamic adjustment of medical service prices.

### Lack of Supportive Policy Environment and Cross-Department Cooperation

Multiple sectors are engaged throughout the entire process of medical service delivery activities. Consequently, the normal functioning of medical service pricing relies on both the rationality of pricing policies and a well-established healthcare system, which is characterized by the supportive policy environment and cross-department cooperation.

One prominent characteristic of such supportive policy environment is reflected as managed competition. Although the healthcare market is characterized by several particularities, there is growing evidence suggesting that competition in the healthcare market would produce better outcomes, which indicates that competition in the healthcare market is more of a positive-sum game rather than the criticized zero-sum game (41–43). However, the principles of fairness, openness, and impartiality fail to be fully complied with in the current healthcare system in China. Compared with public medical institutions, private medical institutions still face a certain degree of unfair treatment in several aspects, including market admission, regular evaluation, supervisory management, professional title assessment, and construction of key specialty departments.

In addition to a supportive policy environment, close cross-department cooperation is also urgently needed, not only between different divisions within the NHSA, but also between NHSA and other governmental sectors. Effective cooperation can significantly contribute to the coordination of price setting, payment methods, and medical insurance system. Otherwise, the normal functioning of medical service pricing will be severely impeded by fragmented administration with poor communications in between different departments.

## Policy Recommendations

Regarding China's ongoing medical service pricing reform, we provide the following policy recommendations.

### Enhancing Governance Capability by Adopting Advanced Methods

On the one hand, optimization of cost accounting process is strongly recommended. The costs of medical service items of different categories, such as treatment services, nursing services, surgical services, and rehabilitation services, should be separately estimated to consider their different characteristics in medical practices. The time-driven activity-based costing could be employed to estimate the specific cost. The average surplus of medical institutions by levels could be used as add-ons to the cost to set the price for a specific service item. The complexity and risk of the services could be considered as the adjusted factors for the price. Moreover, the health technology assessment (HTA) results could also be used as another important reference for the price setting process.

On the other hand, the basic health insurance programs' administrative data, which includes detailed health care information of every insured person, could be utilized to improve the governance capability of medical service pricing administration. The big data analyses should be introduced into every price setting process to explore the “intelligent pricing,” “intelligent regulation,” and “intelligent evaluation.” A rational decision-making rule involving multi-interested parties with multi-criteria should be explored. It is highly recommended that the local government in the region with efficient health information system cooperates with the academic organizations and enterprises to launch a series of pilot programs.

### Advancing Performance-Based Payment to Medical Staffs in Public Hospitals

The performance-based payment in public hospitals should be advanced along with medical service pricing reform, following the “*two permissions*” policy. The first permission of the policy is to allow medical institutions to have the autonomy to adjust wage levels without being limited by the wage-related rules and regulations currently posed on public institutions in China. The second permission ensures the public medical institutions to provide financial incentive for their medical staffs.

It should be noted that the salary of medical staffs should not be directly linked to the revenue of their service provision to avoid possible supplier-induced demand. Instead, the volume of medical services, the degrees of technical difficulty inherent in medical services, the quality of care as well as the operational effectiveness should be adopted to determine the staff's salary, which is a practice of the performance-based payment. In addition, medical institutions are encouraged to use the external hospital evaluation conducted by NHSA and National Health Commission (NHC) for their internal personnel performance evaluation. All these strategies are expected to facilitate the incentive function of medical service pricing, thus ultimately help to establish a reasonable salary system for medical staffs based on the actual value of medical service items delivered in various healthcare settings.

### Constructing a Sound Information Disclosure Mechanism

From a long-term perspective, a nationwide information management platform that performs both information collecting and dynamic monitoring tasks would serve as an indispensable contributor to the effective implementation of the nationwide medical service pricing reform. Meanwhile, an information disclosure system tailored for healthcare-related information has great potential to create incentives for both demand and supply sides of medical services. With dynamic information of healthcare quality and medical expenditures, the information asymmetry issue will be alleviated and the hierarchical medical system will be strengthened from a holistic perspective (44). Based on the real-time information reflective of both healthcare quality and medical expenditures of different service suppliers, medical institutions are inclined to provide medical services of greater values, which would further facilitate the promotion of well-managed competition within healthcare markets. Besides, academic institutions are encouraged to participate in the dynamic evaluation of healthcare-related real-time data to reinforce the effects of social supervision.

## Conclusion

Since the launch of medical service pricing reform, significant breakthroughs have been made with both remarkable improvements and lessons learned. As the pricing reform moves on into a deep-water zone, new insights on medical service pricing are urgently needed to reinforce the implementation of the reform and make further progress. This paper assessed the roles of medical service pricing in China's healthcare system. First, we assessed the potential roles of medical service pricing in China by introducing four policy goals of medical service pricing, including interest coordination, resource allocation, financial compensation, and incentive effect. Our study pointed out that the adoption of medical service pricing approaches served as a significant contributor to the implementation of a wide range of healthcare system goals, which, however, has not received enough attention from policymakers and practitioners. Second, we discussed a list of flaws potentially embedded in medical service pricing policies throughout China's health reform, which implied that the policy goals of medical service pricing have not been fully achieved to produce all the desired outcomes in practice. Third, potential constraints that might compromise the performance of medical service pricing were recognized in multiple aspects, including the excessive diversity and specialization of medical services, price inelasticity of patient's demand, and inadequate capability of both medical institutions and administrations. We then offered a list of recommendations for the ongoing medical service pricing reform: enhancement of governance capability, advancement of performance-based payment in public hospitals, and the establishment of a sound healthcare-related information disclosure mechanism for both quality improvement and public surveillance purposes.

We admitted that this study has several limitations. First, our study was more of a perspective than a rigorous methodology analysis, thus more empirical analyses using data in the future study is needed to further support our arguments. Second, our study failed to conduct case studies to further evaluate each single policy as essential components of the medical service pricing reform. Instead, we provided an overview of medical service pricing policies in China. As such, our future research would focus on in-depth assessment of particular policies to provide further insight into medical service pricing reform in China.

## Author Contributions

JP: study conception and design. YD, YY, and JP: manuscript writing. WX, YZ, and JP: manuscript reviewing and modification. All authors contributed to the article and approved the submitted version.

## Funding

This work was supported by the National Natural Science Foundation of China (Grant Nos. 71874116, 72074163, and 72104159), Ministry of Education of China (Grant No. 20YJC790179), Chengdu Federation of Social Science Association (Grant No. ZZ05), China Postdoctoral Science Foundation (Grant No. 2020M673274), Sichuan University (Grant Nos. 2018hhf-27 and SKSYL201811), and China Medical Board (Grant No. 17-276).

## Conflict of Interest

The authors declare that the research was conducted in the absence of any commercial or financial relationships that could be construed as a potential conflict of interest.

## Publisher's Note

All claims expressed in this article are solely those of the authors and do not necessarily represent those of their affiliated organizations, or those of the publisher, the editors and the reviewers. Any product that may be evaluated in this article, or claim that may be made by its manufacturer, is not guaranteed or endorsed by the publisher.
